# The New Treatment of Rheumatism

**Published:** 1867-06

**Authors:** Chas. E. Buckingham

**Affiliations:** Boston


*The new Treatment of Rheumatism.* MESSRS. EDITORS.—I am glad you published Trousseau's formula from Parrish, as it enables me to correct an error. I had not read it before, but simply saw it was announced as in Parrish's book. Trousseau's own statement is of a syrup saturated with lime. "*Il se prepare en saturant le sirop de sucre par le chaux et en filtrant.*" On looking at Parrish, I find it is to be made of *slaked lime.* This is entirely wrong. *It should be made of caustic lime.* The best formula would be to mix two (2) ounces of lime unslaked and eight (8) ounces of sugar together in the mortar, and pour over the mixture a wine pint of boiling water. Add boiling water enough to mske up the pint, and filter. By the use of boiling water, the operation is more rapid and the formation of lumps is avoided. Of this I have given as as much forty-five drops every two (2) hours in one case of acute rheumatism. Generally, thirty-five (35) drops in half ($\frac{1}{2}$) a tumblerful of milk every three (3) has been enough. The diet in my cases has been left to the patient's choice.

*Boston, March 2, 1867.* Very truly yours,

CHAS. E. BUCKINGHAM.

